# From Prussia to Russia: Russian critics of “Aerztliche Ethik”

**DOI:** 10.18502/jmehm.v12i19.2201

**Published:** 2019-12-31

**Authors:** Boleslav Lichterman

**Affiliations:** *Professor, Department of Humanities, The IM Sechenov First Moscow State Medical University, Moscow, Russia* *.*

**Keywords:** History of medical ethics, Russia, Germany, V. Veresaev, A. Moll

## Abstract

The aim of this paper is to compare “Zapiski Vracha” (“Confessions of a Physician”, first published in 1901) by Vikenty Veresaev to “Aerztliche Ethik” (“Doctors’ Ethics”, first published in 1902; two Russian editions were published in 1903 and 1904) by Albert Moll. It starts with an overview of medical ethics in Russia at the turn of the 20^th^ century in relation to *zemstvo* medicine, followed by reception of Veresaev’s “Confessions of a Physician” by Russian and German physicians, and of Moll’s “Doctors’ Ethics” in Russia. Comparison of these two books may serve as a good example of a search for common philosophical foundations of medical ethics as well as the impact of national cultural traditions.

*A Russian Landscape of Medical Ethics at fin de siècle*

*Zemstva* or elected local self-governing councils emerged in many Russian regions after *krepostnoe pravo* (serfdom of peasants) was abolished in 1861. They were in charge of rural health care and started to build hospitals in the larger villages. A majority of *zemstva *had one physician for every 25,000 - 30,000 people, who lived in areas as large as 25 - 30 square kilometres without proper roads. Apart from working at the hospital (which usually had about 20 beds), a physician had to see between 80 and 100 ambulatory patients daily (up to 20,000 outpatient visits annually). With this patient load a *zemsky* physician would take as little as 2 - 3 minutes to examine a patient. He also had to travel to remote villages to deliver babies, fight epidemics, and supervise the sanitary conditions of 15 - 16 rural schools. The working day of a typical *zemsky* physician lasted for 10 hours. Nevertheless, his average salary of 1200 - 1500 roubles a year was insufficient and would comprise about half of the budget of a middle class family in Germany (1)*. *In such circumstances many doctors were involved in private, fee-for-service practice. 

The trend towards private practice provoked severe criticism from more idealistic colleagues. For example, D. N. Zhbankov claimed that private practice undermines the foundations of *zemstvo* medicine, which is based upon the principle of equal treatment for all. He stressed that “the communal (*obshinnye*) foundations of Russian life, which came to the surface with *zemstvo*, revealed the evil and abnormality of private [medical] practice” (1). Patients’ fees for medical care were condemned as “a tax on the misfortunes of a fellow creature”. Two evils were thought to result from private practice: a waste of time for the society, and the loss of trust among the population. Moreover, Zhbankov objected to any increase in a doctor’s salary: “A workman for the poor and among the poor should not differ from the population from the material standpoint. Otherwise he will not be trusted and his activity will not be productive” (1)^2^.

1. According to the official report of the medical department of the Ministry of Internal Affairs, the total number of medical doctors in Russia in 1881 was 14.488; out of this number, 2629 were private practitioners (18%). In 1907 these figures were 18215 and 5291 (29%) respectively. 

2. As Zhbankov wrote earlier (2), “the patient’s suffering and the doctor’s labor should not be brought to the market and become an issue of demand and supply; honorarium, private practice and all bargains between a patient and a physician must be eradicated, and medical care should be provided free of charge…”. In his article “On the Purpose of Science and Art”, Leo Tolstoy also wrote that true medical assistance will start only when a physician provides it for free and will live among the working people in the same conditions (3).

*Zemskaya* medicine was based upon the principles of free and accessible health care and preventive medicine. These principles were later adopted by Soviet healthcare. According to M. S. Uvarov, yesterday’s slaves treated with mistrust and suspicion everything that originated from their former lords, and this included the suspicion that doctors were poisoning the people (4). Cholera riots during the reign of Nikolas I in 1830s, and the killing of doctors during cholera epidemics in the 1890s serve as vivid examples of these attitudes. “Under such circumstances the Western European pattern of doctor-patient relationships (that is, the relationship between a vendor and a customer) would be difficult to imagine…. There were vendors, but customers were unlikely to come” (4). Most *zemskie* physicians were idealists who viewed their work as public duty. They shared the idea of caring for the people and a sense of indebtedness to the people.

Ethical issues were widely discussed in the medical journals of the late nineteenth century. In just one year, 1894, more than 60 books and articles on medical ethics were published in Russia (5). A weekly periodical, “Vrach” (Medical Doctor) was particularly influential. It was edited by Professor V. A. Manassein (1841 - 1901), who was nicknamed “a knight of medical ethics.” The objectives of this periodical were characterized by Manassein as follows: 

1) To be a true mirror of everything that constitutes real progress in clinical medicine and hygiene;

2) To attract to a cooperative scientific work the maximum number of medical doctors from different regions of Russia; 

3) To provide a constant critical, independent and impartial analysis of all aspects of education, daily life and practice of a medical doctor;

Manassein was strongly opposed to private practice: “The complete trust and purity of the relationship necessary to a doctor for treatment and to a patient for recovery will be impossible unless the doctor’s labor is paid by the society or the state” (5). In 1884 this periodical published a Russian translation of the “Ethical Code of the Warsaw Medical Society” (Warsaw at the time being part of the Russian Empire). According to the editor’s footnote, “if in the future ethical codes for all Russian doctors are going to be elaborated, irrelevant paragraphs have to be modified”. Two sections of the Code were severely criticized: a statement that a physician may breach confidentiality upon the demand of the authorities or when dictated by public interests (article 2), and the obligation of a physician to inform authorities about cases in which he learns about actual or planned criminal activities (article 73). Manassein called for absolute confidentiality in all circumstances. 

He was also against unethical human experimentation. For example, Manassein mentions professor Gübbenet from Kiev who inoculated syphilis into healthy soldiers: “I would like to know if Prof. Gübbenet would inoculate syphilis into his son even if the latter gave his consent” (6). According to Manassein, “no human experiment is permitted unless you are convinced of its complete safety. But even then you should obtain consent from a subject of experimentation” (6). He opposed private practice by university professors for two reasons. First, they have other obligations that they often sacrifice for private practice (we call it conflict of interest nowadays). Second, they deprive of income those physicians who earn money only by private practice. As Manassein’s biographer wrote, “many doctors, especially the old ones, have narrow corporate views. They assume that doctors should be focused on physicians’ interests and even cover the mistakes of their colleagues as if the public and doctors were two enemy camps. Hence there is a special doctors’ ethics. In this regard Manassein was not a physician, but a human being first and foremost, and for this reason he acknowledged human ethics but not corporate ethics” (7).

Sankt-Peterburgskoe Vrachebnoe Obshestvo Vzaimnoi Pomoshi (Physicians’ Society for Mutual Help of St. Petersburg) was established in 1890 for “moral mutual support and fostering of doctors’ unity”. Initially it was supposed to be named the “Society for the Protection of Physicians’ Rights”. This Society organized regular meetings (*Tovarisheskie* *besedy* - Comrades’ talks) to discuss the public aspects of medical life. At the first meeting, held on November 18, 1900, Dr. Evgeny Botkin posed the following question: “Would it be reasonable and possible at all to form a code of medical ethics?” (8)^1^. 

1. See also: Vrach, 1901; 2: 63 - 64

Botkin himself gave a positive answer because he believed this would: 

a) Provide physicians with the opportunity to know the opinions of their colleagues about a particular article of the code; 

b) Help medical doctors gain the trust of the general public, as the latter would learn what things doctors consider to be their obligations; and 

c) Help doctors find out if others have their own professional codes, which is particularly necessary for medical doctors who often treat patients with abnormalities. 

Those who supported the code advanced the following arguments: 

1) An ethical code would assist in resolving difficult or complex cases; 

2) It would constrain weak-willed colleagues; and 

3) It would offer the public a more realistic conception of medical practice. 

During the discussion that followed, the following opposing views were presented: 

1) No code can cover all specific cases, and codification poses the danger that what is not explicitly prohibited will be considered implicitly permissible; 

2) Such a code would hardly reconcile the public with the medical community; 

3) Medical ethics is being treated as a special subject when all the guidance that a doctor needs is a well-known commandment “to love one’s neighbor as oneself”; and 

4) One’s moral instincts speak louder than volumes of ethical codes. 

One of the opponents, Piotr Borisov, his position in the following fashion: “We commit sins not because we do not know a code. Any code that we create could easily serve as a screen sheltering improper actions” (8). The question was eventually put to a vote and the majority of those present voted in favor of the code of medical ethics. 

The questions addressed at the next meeting were: “What is the significance of a code which determines the relations between medical doctors and between doctors and the public?” and “Should such a code be obligatory or just have a moral significance?” It was decided that a code should be obligatory for members of the Physicians’ Society for Mutual Help of St. Petersburg, and have moral significance for non-members. Topics in medical ethics such as a doctor’s obligation to attend a patient upon his or her first request, confidentiality, and how to behave during consultations with other doctors were also discussed. The Society’s by-laws included a Court of Honor “in order to resolve misunderstandings between members and review accusations against members concerning deeds that are blameworthy and incompatible with doctors’ dignity.” The Court of Honor consisted of three members and two candidates who were elected for one year at the annual meeting of the Society. Appeals to the Court of Honor were rare – there were only 16 cases for the 10-year period from early 1890s until early 1900s. (9). According to Dr. Fainshtein, a critic, “A Court of Honor cannot exist [in Russia], because we do not share similar views on doctors’ ethics; this subject is not taught at the university and it is underdeveloped, and only few doctors are participating in medical societies where they might have had a chance to learn more about ethics” ^1^.

1. Courts of honor originated from arbitration courts in the beginning of the 19^th^ century amidst military corporation, which was very concerned with matters of honor, in the motherland of militarism, Prussia, and from there they came to Russia. Gan compared a court of honor to “a shield, which should break waves of calumnies”. According to Gan, medical doctors are trusted by the public because of “the confidence that all dead-wood, everything negative would be eradicated by doctors themselves from their community, that there is a controlling and retributive organ of doctors’ morality” (10). The jurisdiction of a court of was limited to ethical issues dealing with relationships of a physician to science (e.g. quackery), to a patient and to another physician. See also a book by A. - H. Maehle on German medical courts of honor (11). It is interesting to note that courts of honor were revived in the USSR after WWII. In May 1947 there was a joint decree of the Council of Ministers of the USSR and the Central Committee of All-Union Communist Party “On establishment of courts of honor in ministries of the USSR and central governmental agencies” in order to “investigate antipatriotic, anti-government and antisocial actions” (12). Eighty-two courts of honor were established. The most striking example in this regard is a process on Klueva and Roskin by the court of honor of the Ministry of Health in June 1947 (9). After Stalin’s death in 1953 the decree

***Veresaev’s “Confessions of a Physician” and its Critics***

"Zapiski Vracha" ("Confessions of a Physician", also translated as “Memoirs of a Physician”) by Vikenty Veresaev (Smidovich) (1867 - 1945) published in 1901 in a Russian literary periodical “Mir Bozhy” provoked an enormous interest both among the general public and medical community, and triggered discussions on medical ethics. 

Vikenty Vikentievich Smidovich (Veresaev was his pen name) was born in 1867 in Tula, a provincial city 200 km south of Moscow, into a doctor's family^1^. In 1888 he graduated from the philological faculty of St. Petersburg University and decided to study medicine at Dorpat (Yurjev) University. On passing the qualifying examination in 1894, Veresaev began working as a resident doctor at the S. P. Botkin Barracks hospital for the poor in St. Petersburg and at the same time joined a Marxist literary circle. Like Chekhov, Veresaev started to publish short stories and novels, and became a professional writer. In 1901 he was fired from his hospital job by order of the city governor, and by decree of the minister of internal affairs he was prohibited from living in St Petersburg or Moscow for two years. He was mobilized to the Russian-Japanese War where he served as a physician from 1904 to 1905. He wrote many novels and short stories, but considered his best book to be “Zhivaya Zhizn’” (“Alive Life”). The first part, published in 1910, was dedicated to the writings of Fedor Dostoevsky and Leo Tolstoy, and the second part, published in 1914, to Hellenic tragedies and Friedrich Nietzsche. During the last decades of his life Veresaev translated Homer’s “Iliad” and “Odyssey”. He died while editing the last song from “Iliad”.

 Nonetheless, Veresaev is first and foremost known as the author of “Zapiski Vracha” (“Confessions of a Physician”). In the early decades of the 20^th^ century the book had 16 Russian editions and was translated into many languages, including German (eight editions), French, English, and Japanese^2^.

"I am but an average practitioner. I am about to describe my emotions on my first acquaintance with medicine, what I expected of it, and how it actually affected me. I will endeavor to set down all, hiding nothing, and I will strive to write with absolute frankness", Veresaev writes in the introduction (15). In the book he describes his experience of working among the poor. He writes about the unsatisfactory medical education system and discusses medical errors, autopsies and vivisections, private practice, and philanthropy. A separate chapter is dedicated to experimentation on humans, mostly in venereology, because "many questions which, in other branches of medicine, find their answer in experiments on animals can, in venereology, only be decided through human inoculation, and venereologists have not hesitated to take the plunge: crime stains every step taken by their science."(15). Veresaev provides numerous cases of inoculation against gonorrhea, soft ulcer and syphilis in men, women, and children in different countries in the 19^th^ century^3^. 

1. For a short biography of Veresaev in English – see Naomi Raskin’s paper (14).

2. A British edition: Veresaeff V., The Confessions of a Physician (translated from Russian by S. Linden), London: Grant Richards, 1904. An American edition: Veresaev VV., Memoirs of a Physician. New York: Knopf, 1916 (15). All citations from Veresaev’s book are taken from the American edition, which can be downloaded for free from www.archive.org.

3. J. Katz checked all references and confirmed their accuracy in his “Experimentation with Human Beings” published in 1972.

According to the editor of the American edition of Veresaev’s book (Dr. Henry Pleasants, Jr.) “In this country, conditions are not the same as those in Russia twenty years ago [….] Public spirited men and women all over the country are working for the advancement of our profession. Will it alter their viewpoint if they know that ten or fifteen years ago certain science-mad individuals on the other side of the Atlantic inoculated healthy children with syphilis to prove whether or not the disease was contagious in the secondary stage? (15) No”. “The Tuskegee Study of Untreated Syphilis in the Negro Male” will start in Tuskegee, Alabama in 1932.

Veresaev’s book provoked controversial reviews. As a rule, it was highly praised in the press for the general public but severely criticized by medical press. One popular newspaper called the book “a public confession of one for all”. The weekly medical periodical, Vrach, praised the talented young author and the truthfulness of many of his statements. Later a reviewer in the same periodical accused Veresaev of evident exaggerations "that may only bring harm by dissemination of mistaken views in our society, which trusts quacks and medical doctors equally"^1^. 

1. See also comments in Vrach, 1901; 14:459 and 16:528.

The president of the St. Petersburg Medico-Chirurgical Society, Prof. N. A. Vel'yaminov, delivered a speech at the annual meeting in which Veresaev was described as a person with "huge self-importance who is constantly in doubt about his knowledge and his power, indicating an evident egotism with evident nervous irritability. That is why this book is unhealthy" (16).

The success of Veresaev’s book was explained by the fact that a physician echoed thoughts of the lay public about “the essence of medicine”. Many critics accused Veresaev of public revelation of problems and mistakes in medical practice. One of them compared the book to introduction of fine arts to Russian peasants by demonstrating pictures of naked women.

The English translation was critically reviewed in the British Medical Journal (17). An anonymous reviewer wrote, "We find a Russian physician washing his dirty linen in public with every sensational accompaniment that is calculated to attract attention to the nasty business", whereas problems of medical ethics should be discussed only among professionals. "The proper place for Veresaeff's ‘Confessions’ is not the drawing room-table, but the dustbin," concluded the review (17).

Two German physicians also wrote critical pamphlets on Veresaev’s book. These were immediately translated into Russian and published as separate brochures.

 Dr. L. Külz from Leipzig addressed Veresaev in the following fashion: “Your confession is a purely Russian matter. It is so Russian that we, Germans, cannot understand it. Such cases would be impossible for a German physician. But you repeatedly dare to touch issues of international medical importance and quote our German authorities. Thus your writings concern us German physicians. Why do you initiate profanities into our problems?” (18). He underlines that the situation in Germany is different from Russia, because by Russian law a physician is obliged to provide medical help. Külz comments on horrible figures of suicide rates of Russian physicians (3.4% of the total number of Russian medical doctors committed suicide and for *zemskie* physicians this figure rose to 10%). “German physicians do not come across such blind hatred and animosity as their Russian colleagues” he argued. However, he agreed with Veresaev that doctors’ financial situation is very bad, but thought that most German physicians would be happy with the Russian practice of immediate payment from the patients rather than wait to receive money for a job that was done a long time ago. Külz admits that he would prefer that a physician be as well-paid as civil servants, but it would be unthinkable to charge the state with payment of doctors’ honorariums “because then physicians would be equaled to civil servants. And this does not require objections since everyone would understand what harm it might bring” (18). He suggests comparing Russian patients with the German ones who are protected by sickness funds (krankenkassen). 

Külz writes that Veresaev’s comparison of a patient to a clock-work is incorrect. It would be more accurate to compare a patient to a growing tree. A physician is like a gardener who must water his “tree” and provide it with access to the sun, and one who “has a right and even obligation to cut off ill branches and bad spears” (18). With regard to medical experimentations he accuses Vereaev of exaggeration and opening old sores. According to Külz, the main mistake of Veresaev is his fascination with philosophy. He quotes a Latin proverb (“Ne sutor supra crepitam”), and states, “Medicine is an objective science and should not be spoiled by philosophical teachings”. Similar to Vel’jaminov, Külz diagnosed Veresaev as neurasthenic and a “Niktalop, who is blinded by daylight and can orient himself only in the shadow of our science”. Veresaev is called “an ill bird that fouls its own nest”. “Dr. Külz in his answer to Veresaev speaks on behalf not only of German but of Russian physicians as well”, wrote a Russian publisher in his preface to Külz’s brochure (18).

Another critical German brochure was authored by W. von Holst from Riga (19). Von Holst sides with many Russian critics that the problems raised by Veresaev should be discussed *intra muros*. He assumes that a physician can just treat a patient, but only the nature can cure. Doctor-patient relationships are similar to relations between a teacher and a pupil. That is why a patient should strictly follow the doctor’s instructions.

In reply to his critics Veresaev published a pamphlet “Po Povodu ‘Zapisok Vracha’: Otvet Moim Critikam” (“A Propos ‘Confessions of a Physician’: A Reply to My Critics”). According to Veresaev, the relationship between medical science and patient’s personality is a key issue:

“Ethical problems of our profession may not be settled by a tiny codex of professional ethics. Sadly, we should admit that our science does not have ethics yet. One cannot mean by ethics that special corporate doctors’ ethics which just regulates (the) relationship between doctors and (the) public, and between doctors themselves. Ethics in a broad, philosophical sense is needed. Such ethics should cover in full the above-indicated problem of the relationship between medical science and a living personality” (20).

Veresaev argues that a problem “of borders beyond which (the) interests of an individual might be sacrificed to the interests of science […] is not a specific problem of some special doctors’ ethics, but a great, eternal, fundamental problem of the relationship between personality and higher categories such as society, science, law etc.” (20). As Veresaev writes, “Narrow problems of medical practice *first and foremost* should be resolved from the philosophical standpoint” (20).

***Russian Critics of “Aerztliche Ethik”***

In 1902 Physicians’ Society for Mutual Help of St. Petersburg started to publish its periodical “Vestnik Sankt-Peterburgskogo Vrachebnogo Obshestva Vzaimnoi Pomoshi”. The first paper in the first issue of this periodical recommended that Society members and readers pay attention to the “gigantic work by Albert Moll, ‘Aerztliche Ethik’ (Doctors' Ethics), which was just published” (21).

Moll’s book was extensively reviewed in leading Russian medical periodicals. A weekly periodical entitled “Russky Vrach” *(“Russian Medical Doctor” – a successor of “Vrach”, printed from 1902 to 1918) *published a review by Piotr Borisov^1^, who stressed the importance of Moll’s book for Russian physicians. Borisov provided a brief overview of the problems covered by the book under review and concluded that "the author did not find a solid foundation of doctors' ethics in any [philosophical] system and founded it upon everyday practice" (22).

1. Piotr Yakovlevich Borisov (1864 - 1916) was the son of a merchant. He graduated from the Military Medical Academy in St. Petersburg in 1889 cum eximia laude and worked there at a chair of physiology. His doctoral thesis defended in 1891 was dedicated to pepsin properties. In 1895 he was elected privat-docent (associate professor) at this chair and worked under Ivan Pavlov from 1895 to 1903. In 1903 he was elected a chair of pharmacology and balneotherapy (ordinary professor since 1904) of Novorossiysk University in Odessa where he stayed until his death from stroke in 1916. He authored more than 40 scientific publications. Being a member of the party of constitutional democrats (*Kadety*) he actively participated in political life during elections to Russian parliament (*Gosudarstvennaya Duma*).

He considered this foundation as shaky since Moll himself admitted that the same action might provoke different feelings among different people, and moral feeling is often influenced by traditions and etiquette. The reviewer disagreed with the author that ethics cannot be based on the theory of general progress since treatment supports the lives of disabled and weak persons. Physical strength could not be viewed as a core of progress: “A sick writer would do for general progress more than the strongest and healthiest manual worker”. Another example is an idiot who provokes feelings of compassion in ordinary people. According to Borisov, ethics is based on two laws: 1) continuation and preservation of the human race; and 2) preservation of the life of every living being. 

“Vestnik Sankt-Peterburgskogo Vrachebnogo Obshestva Vzaimnoi Pomoshi” published a lengthy, 9-page critical review of Moll’s book by N. G. Freiberg^1^. 

1. Nikolai Gustavovich Freiberg (1859 - 1927) was born in a family of intellectuals in St. Petersburg where he studied at Petri-Schule (a famous German gymnasium). He graduated from Military Medical Academy in 1883 and worked as a surgeon at a military hospital until 1892 when he joined the medical department of the Ministry of Interior. He was appointed a chief of administration of the Commission on Revision of Medical and Sanitary Laws. Freiberg was elected a co-editor of “Vestnik Sankt-Peterburgskogo Vrachebnogo Obshestva Vzaimnoi Pomoshi” from 1904 to 1906. He represented Russia at the International Sanitary Conferences for almost 10 years (from 1908 to 1917) and was a national delegate at the Permanent Committee of the International Bureau of Public Hygiene. In 1918 he was appointed a chief of administration of the Russian Ministry of Health (*Narkomzdrav*). He suggested a new branch of hygiene named administrative hygiene and coauthored “A Short Textbook of Hygiene”. 

He wrote that Moll erroneously applied the term ‘ethics’ to practical problems such as doctors' obligations to the public, to colleagues, to the state: "All these problems might be covered by teaching about obligations of a physician - by medical deontology" (23). Although deontology is based upon ethical grounds, it is not ethics. Ethics is a science about the laws of manifestations of moral feeling and is the same for all professions. Thus the term ‘doctors' ethics’ represents a logical contradiction.

 Feinberg is also critical of the concept of a tacit contract between doctor and patient advocated by Moll. Moll’s arguments are labeled as scholastic, and he mixes up the principal problem and its practical consequences. Feinberg concludes, “If you throw out of Moll’s book elements of abstract ‘doctors’ ethics’, there will be some very interesting reasoning on different problems of doctors’ activity” (23). 

The third detailed review of Moll’s book was published in 1903 in “Bol’nichnaya Gazeta Botkina” (Botkin’s Hospital Newspaper) by V. B-kov and was focused on the interplay of doctors’ ethics and medical care (24). Limitation of doctors’ ethics to *bona fide* execution of doctors’ responsibilities is considered as a one-sided approach. Moll mistakenly declares that doctors’ ethics is interesting for physicians only whereas in real life non-physicians are also working in this domain. For example, Russian lawyer and senator A. F. Koni wrote on the subject of confidentiality and became a recognized authority in medical ethics. 

The reviewer also mentions Neisser’s medical experiments criticized by Moll. On the other hand, Moll considers it unjust to rebuke some personalities when the whole system requires radical change. The reviewer disagrees with Moll on many occasions. It is impossible to plan a doctor’s actions in all cases of his or her practice. Doctors’ ethics should be based on general human ethics: “Morals is universal. In doctor’s activity it might be diversified but it might not be changed” (24). The review ends with critical remarks addressed to “Confessions of a Physician” by Vikenty Veresaev which shook fundamental morals and was especially harmful for female students. The reviewer is convinced that Moll was also impressed by Veresaev’s book but could not digest it and stand above it.

There were two Russian translations of Moll's book. The Moscow edition was edited by V. Veresaev, who also provided a critical preface (25). Veresaev pointed out the major shortfalls of “Aerztliche Ethik”, stating that the main “external” shortfall is the “extreme circumlocution and ample style” of Moll’s book (26). “Dr. Moll meticulously communicates his thoughts that would be more suitable for copy-books for schoolchildren rather than for a serious work on ethics” (26). But more important is the “internal” shortfall due to Moll’s mentality. Vereasev accused Moll of philistinism: "Everywhere, as soon as Moll goes beyond purely medical ethical matters, we can see a cautious, moderate and prudent philistine, who is devoid of noble purpose, without a wide range of interests, whose only ideal is to honestly earn a piece of bread for himself and his people" (26). In accordance with the author’s spiritual outlook, individual names are almost lacking in “Aerztliche Ethik”. For example, Moll condemns human experimentation and provides a lengthy list of such experiments during the last years. But “nomina sunt odiosa” according to Moll’s “practical morals” and, Veresaev notes, “the careful author writes about these experiments in the following manner: ‘*One* physician in *one* municipal hospital performed such (an) experiment’. This spirit of deep petit bourgeois philistinism comprises the most repulsive side of Moll’s work. Luckily, this spirit is mostly alien to the Russian medical community and we hope that at least some of our readers would forgive us for omitting in this translation extensive deliberations by Moll which are of interest solely as material for characteristics of bourgeois outlook of the modern ordinary German physician” (26). That is why the chapter about the physician’s private life was omitted in this Russian edition.

 Despite all the above mentioned shortcomings, Moll’s book has several advantages. “The book by Dr. Moll is a book about *real* doctors’ ethics. That is its main advantage” (26). Another big advantage is the book’s human approach. When dealing with ethical problems, a physician should stand above the narrow professional standpoint. For example, Vereasev mentions “an exemplary paragraph on confidentiality”, which is devoid of the schematism of professional ethics. According to Veresaev, preservation of confidentiality should be counterweighted by the other human and civil duties of a physician.

 The chapter on risky medical procedures is called “one of the best chapters of the book”. But Veresaev objects to Moll’s view that the human fetus is not a human being. He argues that a human being appears at the moment of conception and equals termination of pregnancy (abortion) to homicide.

A St. Petersburg edition of Moll's book was translated with a commentary by Dr. Ya. L. Levenson and had a revealing subtitle: "For medical doctors and general public" (27). As Levenson noted in his preface, the art of healing is imperfect and for this reason a physician should have a very high level of bona fide moral purity in order to do everything possible for a patient, or at least to avoid inflicting any harm. These qualities are especially important for Russian physicians due to the deficiencies in our social fabric such as poverty and lack of enlightenment of the people: “(the) Russian physician should understand that he is not only a physician strictu senso but also a citizen […] and his task is not only to prescribe a drug or treat a disease of a patient, but also to enlighten and support him” (28). However, there are often fierce but fruitless debates on problems of medical ethics. According to Levenson, such situations can be explained by the lack of philosophical foundations of ethical judgments. The aim of Moll’s book is to provide these foundations both to the public and medical doctors. “Aerztliche Ethik” is the most complete publication on medical ethics and it clearly distinguishes pure ethical concepts from problems pertaining to doctors’ etiquette and medical politics. Another advantage is the author’s impartiality. Moll is not afraid of being accused of washing his dirty linen in public.

The German edition was aimed mostly at medical doctors. For the Russian edition, Levenson excluded everything of local (German) interest. Numerous footnotes were added and all special terms were replaced by words in general use in order to make Moll’s book accessible for public. The book has a supplement on public health in Russia written by M. S. Uvarov (see above) and based on *zemstvo* medicine (4). As Levinson noted, “under the close links that exist between physicians and general public, mutual mistrust and misunderstanding might be removed only in case that both sides would be acquainted with their moral rights and obligations” (28).

## Conclusion

At the turn of the last century we see a considerable interest in problems of medical ethics both among the general public and medical community. These problems were approached differently in Russia and in Germany due to the different social and political contexts. In Russia the ideas of freely accessible medical care were rooted in *zemstvo* medicine, whereas in Germany medical doctors were primarily private practitioners. Comparison of Veresaev’s “Confessions of a Physician” to Moll’s “Doctors’ Ethics” may serve as a good example of the search for the common philosophical foundations of medical ethics as well as the impact of national cultural traditions. The discussions provoked by these two books are also relevant for modern bioethical discourse.

## Notes:

**Citation to this article:**

Lichterman B. From Prussia to Russia: Russian critics of “Aerztliche Ethik”. J Med Ethics Hist Med. 2019; 12: 19.
